# Supplementary material for: A systematic review of federated learning applications for biomedical data
Source: PLOS Digit Health. 2022 May 19;1(5):e0000033. doi: 10.1371/journal.pdig.0000033 (PMC9931322; doi:10.1371/journal.pdig.0000033)
Supplement: S2 Table — (DOCX) [file pdig.0000033.s002.docx]

**SUPPLEMENTAL Table 2.** Systematic review search strategy.

| **Search Recipe** | **Number of Manuscripts** |
| --- | --- |
| ‘Artificial intelligence’/exp OR ‘machine learning’/exp OR ‘machine learning’:ti,ab OR (Federated Near/3 (database OR network OR learn* OR environment)):ti,ab OR (distribut* Near/3 (learning OR comput*)):ti,ab | 242,464 |
| (‘multicenter’ OR ‘multi-center’ OR ‘Multi-site*’ OR ‘multi-institut*’ OR ‘cross institut*’ OR decentralize* OR ‘Privacy preserv*’ OR ‘Data exchange’ OR ‘mutli-party comput*’):ti,ab OR ((multi*) Near/3 (site OR institut* OR data*)):ti,ab OR ((sharing) Near/3 (information OR Data OR research)):ti,ab OR ((research Near/3 (Collab*)):ti,ab) | 327,599 |
| ('electronic health record'/exp OR 'medical informatics'/exp OR 'electronic medical record'/exp) OR (biomedical research OR EMR OR EHR):ti,ab OR ((health or hospital or medical or patient*) Near/3 record):ti,ab OR ((clinical or research or health or hospital or medical or patient) Near/3 Data):ti,ab OR ((medical OR health OR patient*) Near/3 information):ti,ab | 729,814 |
| **Distinct Publications** | 2,173 |
