# Supplementary figures and images for: A systematic review of federated learning applications for biomedical data
Source: PLOS Digit Health. 2022 May 19;1(5):e0000033. doi: 10.1371/journal.pdig.0000033 (PMC9931322; doi:10.1371/journal.pdig.0000033)

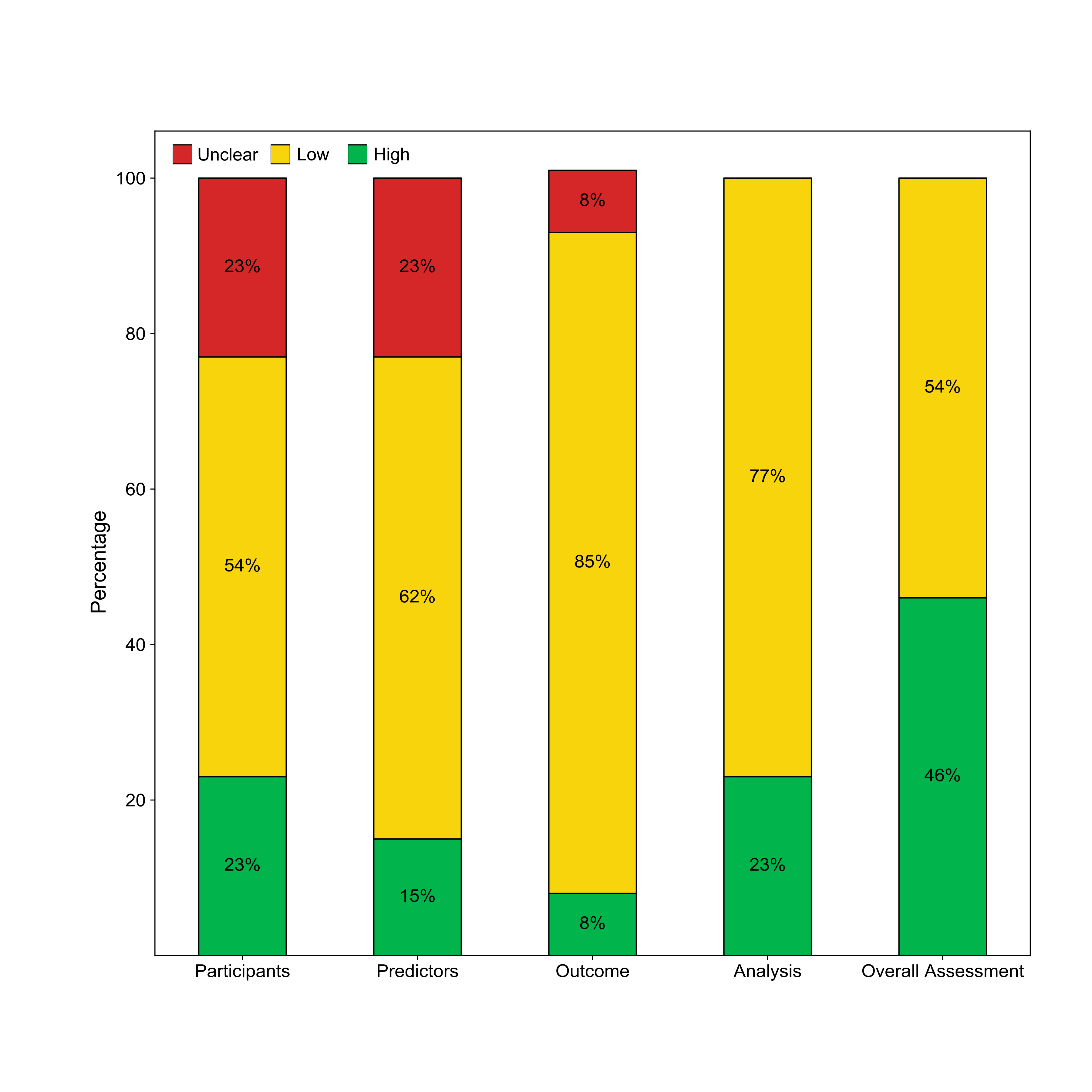

Supplement: S1 Fig — (TIFF) [file pdig.0000033.s005.tiff]
