# Supplementary material for: A systematic review of federated learning applications for biomedical data
Source: PLOS Digit Health. 2022 May 19;1(5):e0000033. doi: 10.1371/journal.pdig.0000033 (PMC9931322; doi:10.1371/journal.pdig.0000033)
Supplement: Attachment — Submitted filename: PLOSdigitalhealth Rebuttal ltr 3.15.22.docx [file pdig.0000033.s007.docx]

**Matthew G. Crowson, MD MPA MASc FRCSC**

Attending Surgeon, Otology & Neurotology, Department of Otolaryngology-Head & Neck Surgery, Massachusetts Eye and Ear

Investigator, MEE Jenks Vestibular Laboratory

Member of Faculty, Department of Otolaryngology-Head & Neck Surgery, Harvard Medical School

Clinical Associate, Massachusetts General Hospital

March 15 2022

To the *PLOS Digital Health* Editorial Committee,

We thank the editorial staff and reviewer for their thoughtful comments and critiques of our original research/systematic review article titled “***A Systematic Review of Federated Learning Applications for Biomedical Data.***”

As per the instructions, we have responded to each point raised by the editor and reviewer below:

Journal/Editorial Comments:

*1. Please amend your detailed Financial Disclosure statement. This is published with the article, therefore should be completed in full sentences and contain the exact wording you wish to be published.*

We have amended the financial disclosure statement to read **“The funders had no role in study design, data collection and analysis, decision to publish, or preparation of the manuscript.”**

*2. Please update your Competing Interests statement. If you have no competing interests to declare, please state: “The authors have declared that no competing interests exist.”*

We have amended Competing Interests statement to read **“The authors have declared that no competing interests exist.”**

*3. Please provide a complete Data Availability Statement in the submission form, ensuring you include all necessary access information or a reason for why you are unable to make your data freely accessible. Note that it is not acceptable for the authors to be the sole named individuals responsible for ensuring data access.*

The data used for this systematic review is derived from publicly available manuscripts. We will emphasize this point further in the Data Availability Statement:

**“The data for this systematic review is derived from a qualitative assessment of publicly available published papers/manuscripts. The manuscripts referenced in this paper are in the public domain.”**

*4. Please provide separate figure files in .tif or .eps format only and ensure that all files are under our size limit of 20MB.*

We have uploaded the figures as separate files in .TIF format.

Reviewer #1:

*1. " Although the scope of the systematic review is defined in the beginning, I am missing some more remarks regarding the natural privacy (or lack thereof) of federated learning. Recent works have shown the vulnerability of FL to reconstruction attacks (e.g. link redacted) and other kinds of attacks, which is not mentioned at all in the review. Due to the possible leakage of sensitive patient information using FL without measures such as Differential Privacy, to me this topic is essential to mention somewhere in the review.”*

We thank the reviewer for their comments and suggestions for strengthening our manuscript.

The reviewer makes the astute observation that while federated learning is designed, in part, to mitigate data privacy risks it is still subject to adversarial attacks and other data loss risks. We agree that it is worth commenting on this topic as a critical area for consideration for further work in this space. To the discussion we have added this section:

**Federated Learning & Privacy**. While FL allows ML model building without raw data collection from multiple institutions, the possibility for an adversary to learn about or memorize sensitive user data by simply tweaking the input datasets and probing the output of the algorithm exists [1,2]. Differential privacy (DP) is a new notion that is tailored to such federated settings capable of providing a quantifiable measure of data anonymization [3]. Several techniques are being explored such as distributed stochastic gradient descent, local and meta differential privacy methods [4,5]; which essentially adds noise to preserve the user-data privacy during the federated training. While there is much focus on privacy in FL, note that there is a crucial tradeoff between the convergence of the ML models during training and privacy protection levels as better convergence comes with lower privacy. Further research on privacy-preserving FL architectures with different tradeoff requirements on convergence performance and privacy levels is therefore much desirable.

Added References:

[1] Shokri, R., Stronati, M., Song, C., and Shmatikov,V. (2017). Membership inference attacks against machine learning models. In 2017 IEEE Symposium on Security and Privacy (SP), pages 3–18. IEEE.

[2] Fredrikson, M., Jha, S., and Ristenpart, T. (2015). Model inversion attacks that exploit confidence information and basic countermeasures. In Proceedings of the 22nd ACM SIGSAC Conference on Computer and Communications Security, pages 1322–1333.

[3] K. Wei *et al*., "Federated Learning With Differential Privacy: Algorithms and Performance Analysis," in *IEEE Transactions on Information Forensics and Security*, vol. 15, pp. 3454-3469, 2020, doi: 10.1109/TIFS.2020.2988575.

[4] J. Li, M. Khodak, S. Caldas, and A. Talwalkar, “Differentially Private Meta-Learning, ” arXiv, 2019. [Online]. Available: [https://arxiv.org/abs/1909.05830](https://secure-web.cisco.com/1b6Mcx2iSN4hBMtvOqHbNfaADw1sOBejiGw5X-jnwufiIhTUNdy-leLYC_MRr0fUimty7KRrivzo4wXyEVJ1ho51D_ppfeOISI46xRVY-LAPMEAJtDHjfKClDSv3K-sLYaXwtom5HAHYDzggc3TKq0nYq6JDkkBBzyLoV7MhgYG0Bp4U3Hh7EH_Y9YHYOjJ_-TjfA_mTyG7c2TNa_5bnMZE8STzxgwOtzi0li2gcMuKF7aic7qBQuetSOgkfcfNxnDOupj0NNsAgQWaIFlbep4WbBkezeSkYxWrs88haCJybYdRGzJEEr8fPrKZqcluNl/https%3A%2F%2Farxiv.org%2Fabs%2F1909.05830)

[5] R. C. Geyer, T. Klein, and M. Nabi, “Differentially Private Federated Learning: A Client Level Perspective,” arXiv, 2017. [Online]. Available: [http://arxiv.org/abs/1712.07557](http://secure-web.cisco.com/1PP60XcITCVoSEcEr2Y3kFWDyAZGmxzqnIOYvE3PwqvlN58va9b8Qd4UgY_mui4oC6rBf3DJkF_TTEdbZ11HKfesy_jYZO7XrCDTAfnU2aoAi8xcjuL0MSUoMY6wzhjYjraWrUjTLPAIaEDEopbGNbtO8LItuIxKG7F4Lajl3c1Oa2uc-b82sM4XOsY0bDnvmW_2l9IicaZTSIqNzAd1YHqhBPTOHioRPLPrXKwixgbsm1UZ5JoqQiPAG9JhDeU3zXVUJVqyQkOfv6kCpeoEMlYh-zYEzwhEvuuCZ7gMHaNgukayE6__5o6Lm9Km6QqaP/http%3A%2F%2Farxiv.org%2Fabs%2F1712.07557)

*2. “The paper could also benefit from mentioning and briefly discussing existing systematic literature reviews in this field (e.g. link redacted) explaining the unique value of the author's review in comparison. I understand that this does not fit into the Results section of the paper, but could be included in the Discussion.”*

The reviewer is correct that several review papers exist that review federated learning in a biomedical context (several structured as informal narrative reviews, however and not formal systematic reviews) and even still some have come to light as late-breaking late in 2021 that escaped our attention as we submitted our manuscript for publication consideration.

We agree we should acknowledge the co-existence of other formal systematic reviews in context of what our manuscript adds to the larger knowledgebase on the topic. On our review of these other works, we feel our paper is unique in the focus on the clinical applicability of FL as opposed to an emphasis on comparison and contrast of the nuances of FL architectures themselves.

**“Prior narrative and systematic reviews on FL as applied to healthcare have elaborated on the technical nuances of FL architectures, models, and datasets as well as higher order issues such as legal contexts, privacy, and ethical considerations. [Zerka 2020; Pfitzner 2021; Shyu 2021]. In our systematic review, we add to this existing knowledge by evaluating the current state of FL in biomedical contexts through a search of studies reporting on ML algorithms developed and validated using a FL framework specifically for biomedical data.”**

**Added references:**

Pfitzner, B., Steckhan, N. and Arnrich, B., 2021. Federated learning in a medical context: A systematic literature review. *ACM Transactions on Internet Technology (TOIT)*, *21*(2), pp.1-31.

Shyu, C.R., Putra, K.T., Chen, H.C., Tsai, Y.Y., Hossain, K.S.M., Jiang, W. and Shae, Z.Y., 2021. A Systematic Review of Federated Learning in the Healthcare Area: From the Perspective of Data Properties and Applications. *Applied Sciences*, *11*(23), p.11191.

Zerka, F., Barakat, S., Walsh, S., Bogowicz, M., Leijenaar, R.T., Jochems, A., Miraglio, B., Townend, D. and Lambin, P., 2020. Systematic review of privacy-preserving distributed machine learning from federated databases in health care. *JCO clinical cancer informatics*, *4*, pp.184-200.

*3. “In the section "Methods - Search Strategy & Selection Criteria", the searched literature collections are listed. To me it is not apparent why those specific libraries were selected, and not for example IEEE Xplore. If there is some reasoning behind this, it would be good to include it here.”*

For the purposes of a biomedical systematic review, it has been the tradition to search at least two of the three main English-language biomedical publication repositories (i.e., CINAHL, Ovid-MEDLINE, EMBASE) given the clinical content focus. We agree that our search strategy may have been enhanced if we included other repositories such as *IEEE Xplore* or other non-biomedical technical paper repositories. We will certainly use this feedback moving forward in our subsequent study of this topic.

*4. “The table captions do not always explain the table sufficiently. For instance, in Table 2, I do not understand the meaning of the column “Number of Entries” compared to the column “Patient Cohort Size”. Please clarify in the table caption or rename the column appropriately.”*

We agree that the “**Number of Entries”** column header is vague and confusing when compared to “**Patient Cohort Size**.” We intended to include both how many patients’ data’ were included in the papers, as well as the dataset size used for modeling *derived* from those patients.

We have amended the latter column to read “**Modeling Dataset Size”**

*5. “In Figure 1 for the number entry for “Records after duplicates removed”, the authors used the PRISMA flowchart slightly incorrectly. I believe the number n corresponds to the number of papers still included after duplicate removal, which would be 2171, as in the box for “Records screened”.”*

Thank you for identifying this error. We have amended figure 1 to properly identify the number of duplicates removed as a branch of the total remaining papers.

*6. “In Table 2 for the “Number of Entries” for “Huang et al.”, the thousands separator is shifted one digit to the left.”*

Thank you for catching this typographical error. It has been corrected.
